# From Nutrient to Nanocarrier: The Multifaceted Role of Vitamin B12 in Drug Delivery

**DOI:** 10.3390/ijms26115119

**Published:** 2025-05-26

**Authors:** Nikita A. Kuldyushev, Sergey Y. Simonenko, Semen I. Goreninskii, Tatiana N. Pallaeva, Andrey A. Zamyatnin, Alessandro Parodi

**Affiliations:** 1 Research Center for Translational Medicine, Sirius University of Science and Technology, 354340 Sochi, Russia; 2 Additive Technologies Center, Tomsk Polytechnic University, Lenina av., 30, 634050 Tomsk, Russia; 3 A.V. Shubnikov Institute of Crystallography of the Kurchatov Complex Crystallography and Photonics of the NRC “Kurchatov Institute”, 119333 Moscow, Russia; 4 Life Improvement by Future Technologies (LIFT) Center, 121205 Moscow, Russia; 5 Department of Biological Chemistry, Sechenov First Moscow State Medical University (Sechenov University), 119991 Moscow, Russia; 6 Belozersky Institute of Physico-Chemical Biology, Lomonosov Moscow State University, 119992 Moscow, Russia; 7 Faculty of Bioengineering and Bioinformatics, Lomonosov Moscow State University, 119234 Moscow, Russia

**Keywords:** vitamin B12, drug delivery, nanomedicine, neurological conditions, cancer, nanoparticles

## Abstract

Vitamin B12 (B12), a crucial water-soluble vitamin, plays an essential role in various cellular functions, including DNA synthesis and cellular metabolism. This review explores recent advancements in B12 delivery systems and their potential applications in drug delivery. The unique absorption pathways of B12, which involve specific binding proteins and receptors, are highlighted, emphasizing the vitamin’s protective mechanisms that enhance its bioavailability. The review discusses the intricate multi-protein network involved in B12 metabolism and the implications of B12 deficiency, which can lead to significant health issues, including neurological and hematological disorders. Additionally, the potential of B12 as a drug carrier to improve the pharmacokinetic properties of poorly bioavailable medications is examined. The findings suggest that optimizing B12 delivery could enhance therapeutic outcomes in nanomedicine and other clinical applications.

## 1. Introduction

As the largest and most structurally complex water-soluble vitamin, vitamin B12 (B12) plays a crucial role in human health but can only be obtained through food or supplements. B12 represents an essential nutrient characterized by a distinct digestion/assimilation pathway that safeguards it from degradation during absorption and distribution. After ingestion, B12 binds specific proteins that protect it from the harsh conditions of the gastro-intestinal tract [1].

Once absorbed, B12 quickly binds to B12-binding proteins in the bloodstream and it is selectively transported into cells, where it interacts with various B12-processing proteins [1].

**Figure 1:**
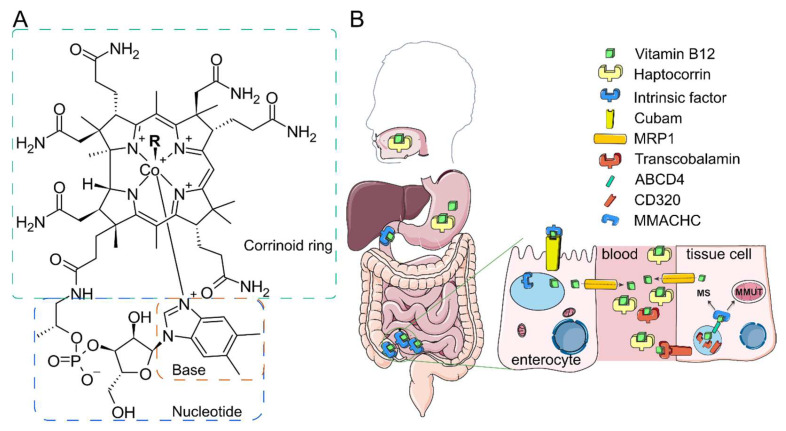
Unique route of B12. (**A**) B12 structure. B12 or cobalamin differ with respect to upper ligands. R designates the upper ligand. (**B**) B12 transport starts in mouth, followed by stomach binding to haptocorrin in the stomach and to intrinsic factor in the duodenum. In the ileum, B12 complex with the intrinsic factor is endocytosed by enterocytes via the cubam receptor. IF releases B12 in the lysosomal acidic pH. Free B12 is transported to the enterocyte cytoplasm and exported to the blood via multidrug resistance protein 1 (MRP1). In the blood, B12 is mainly bound to haptocorrin and transcobalamin (TC). B12 complex with TC can be endocytosed by cells via CD320 receptor. After proteolytic and acidic digestion of TC, free B12 is transferred to the cytosol via ATP-binding cassette transporter ABCD4. MMACHC binds B12 and delivers it either to methionine synthase MS (MS) or to MMAB protein in the mitochondria. MMAA and MMAB together bring B12 to methylmalonyl-CoA mutase (MMUT). Some steps are omitted for clarity. The figure is adapted with permission from [4].

B12 is a generic term for cobalamins, which are corrinoids with an attached nucleotide. Structurally, cobalamin (Cbl) consists of a corrinoid ring with seven amide chains, a cobalt atom coordinated by the central nitrogen atoms of the ring, and a nucleotide covalently attached to one of the amide chains (Figure 1A). The ligand bound to the cobalt atom in the upper plane determines the specific form of B12. Among them, the following ones are included: methylcobalamin (MeCbl, -CH3), adenosylcobalamin (AdoCbl, -adenosyl), hydroxycobalamin (-OH), and cyanocobalamin (-CN) (Figure 1A). In nature, B12 is produced by several bacterial species, such as *Pseudomonas denitrificans* and *Bacillus megaterium*, via aerobic and anaerobic pathways, respectively [2]. Certain microbial residents of the human digestive tract are capable of synthesizing B12 and other corrinoids, influencing the composition and dynamics of the gut microbial community [3]. The de novo biological synthesis of B12 is a complex process involving more than 30 genes to complete the entire biosynthetic pathway. Given its protective mechanisms during absorption and selective transport into cells, vitamin B12 could serve as an effective drug carrier, particularly for medications that require improved oral bioavailability. In the field of drug delivery, challenges such as low bioavailability and rapid degradation of therapeutic agents necessitate innovative solutions. Vitamin B12’s inherent properties may offer promising strategies in overcoming these challenges. This review aims to elucidate the biochemical functions of vitamin B12 while exploring its promising role as a drug delivery vehicle and as payload in nanomedicine, ultimately contributing to advancements in the field.

## 2. B12 Biochemistry

### 2.1. Vitamin B12 Trafficking: From the Gastro-Intestinal Tract to the Blood

B12 can be absorbed from various foods, including meat, milk, and other animal-derived products where B12 is predominantly present in protein-bound forms. Normal absorption of B12 through enterocytes is approximately 1.5–2 µg per day. The absorption of B12 begins in the oral cavity, where small amounts of free, non-protein-bound B12 bind to haptocorrin (Hc), a glycoprotein secreted by salivary glands into saliva at concentrations of several nmol/L [5].

In the stomach, gastric proteases and acidic pH facilitate the release of corrinoids from food proteins that can bind to Hc. Additionally, intrinsic factor (IF), another B12-binding protein, is secreted in the stomach, but its interaction with B12 is affected in this compartment due to acidic pH. In the duodenum, pancreatic proteases digest Hc-bound B12. In the ileum, the B12-IF complex interacts with its receptor cubam, a protein complex composed of cubilin and amnionless, located on the plasma membrane of enterocytes [6]. Small amount (<10%) of B12 is absorbed in colon via other mechanisms [7,8,9].

Following endocytosis of the B12-IF complex into enterocytes, IF loses its affinity for B12 due to the acidic pH and the presence of cathepsin L in the lysosomes [10]. Free B12 is transported into the cytosol via the ATP-binding cassette (ABC) transporter ABCD4 [11], and then it is exported to the extracellular space (including blood) thanks to multidrug resistance protein 1 (MRP1) [12].

B12 transport mechanisms are leveraged to improve stability against gastric proteases and oral bioavailability of therapeutic peptides [13].

### 2.2. Vitamin B12 Trafficking: From the Blood to the Cells

In the blood, B12 is primarily bound by two proteins: transcobalamin (TC) and Hc (Figure 1B). While Hc binds B12 and its analogs, such as cobamides, it may sequester inactive B12 analogs in circulation [14,15].

The glycoprotein TC transports B12 in the blood and this complex enters cells through endocytosis mediated by CD320 (Figure 1B), a heavily glycosylated glycoprotein also known as TC receptor [16]. In the lysosomes, TC loses its binding capacity due to the acidic pH of these vesicles and the action of cathepsins B and L [10,17]. ABCD4 facilitates the efflux of B12 from the lysosomes to the cytosol [11]. B12 is likely transferred directly from ABCD4 to the cytosolic enzyme methylmalonic acidemia and homocystinuria type C protein (MMACHC), which binds both ABCD4 in vitro, preventing dilution or degradation of B12 in the cytosol [18]. MMACHC removes the upper ligand of cobalamins via its decyanase and dealkylase activities. Following this step, methylmalonic acidemia and homocystinuria type D protein (MMACHD) act as adapters for MMACHC, facilitating the transfer of cobalamin to its final destinations, namely methionine synthase (MS) in the cytosol or its mitochondrial transporter methylmalonic aciduria type A protein (MMAA) [19]. In mitochondria, the enzyme MMAB transfers an adenosyl group from ATP to cobalamin to generate AdoCbl [20]. The G-protein MMAA ensures proper adenosylation of cobalamin and facilitates the transport of AdoCbl to methylmalonyl-CoA mutase (MMUT) [21].

Excessive, unprocessed B12 is exported from the cytosol to the extracellular space, with a fraction remaining attached to the cell membrane [22]. In vitro studies indicated that intracellular concentrations of total B12 depend nonlinearly on extracellular B12 levels, ranging from 0.07 to 0.15 nmol per gram of protein with physiological extracellular B12 concentrations (~1 nM) [23]. The retention of B12 is supported by mechanisms that reduce the loss of this scarce nutrient, including renal reabsorption via the megalin receptor [23] and enterohepatic circulation [24]. In humans, B12 is primarily stored in the liver, followed by the kidneys [25].

### 2.3. Vitamin B12 Biochemistry

In the cytoplasm, free vitamin B12 is converted into its active forms MeCbl and AdoCbl that are fundamental for epigenetics and DNA synthesis (methyl donor and nucleotide synthesis) and detoxification in mitochondria, respectively. For this reason, B12 accumulates preferentially in rapidly proliferating cells, both in physiological (i.e., embryonal growth) and pathological (i.e., cancer) conditions.

#### 2.3.1. B12 as a Cofactor of Methionine Synthase

In human cells, vitamin B12 acts as a cofactor for MS [26], where MeCbl facilitates the conversion of Hcy to methionine with N5-methylTHF as the methyl donor [26]. During this process, methylTHF donates its methyl group and is subsequently converted to tetrahydrofolate (THF) [26]. The methionine produced by MS serves as a substrate for S-adenosylmethionine (SAM) synthetase, which converts methionine and S-adenosylhomocysteine (SAH) into SAM—a universal methyl donor in cellular processes [27].

**Figure 2:**
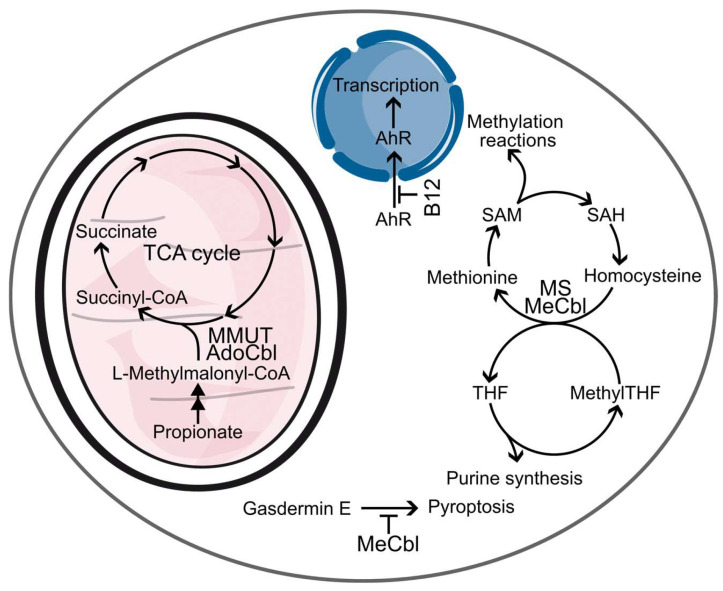
Functions of vitamin B12. B12 participates in several biochemical pathways. MMUT utilizes AdoCbl as a cofactor to convert toxic L-methylmalonyl-CoA into succinyl-CoA and deliver it into the TCA cycle. MS uses MeCbl to catalyze conversion of homocysteine into methionine. Methionine is used to produce SAM, a universal methyl donor. After the removal of the methyl group from SAM, the resulting SAH is enzymatically converted to homocysteine, which is re-used as a source for methionine. To regenerate Cbl to MeCbl in the methionine cycle, nethyl group from methylTHF is used. The lack of MeCbl results in a so-called methyl folate trap, when methylTHF cannot be converted to THF, thus inhibiting purine synthesis. Non-enzymatic functions of B12 include inhibition of gasdermin E activation, preventing pyroptosis, and antagonizing AhR, preventing its transcription activity. Some steps and enzymes are omitted for clarity.

Decreased MS activity disrupts transsulfuration and folate metabolism (Figure 2), leading to increased Hcy levels [28] and reduced levels of SAM [29], THF [30], and the SAM/SAH ratio [31], which can further inhibit MS by SAH [32]. Hyperhomocysteinemia is a significant inflammatory factor [33]. Additionally, a lack of methyl group donors disrupts DNA and histone methylation processes [34]. Factors contributing to reduced MS activity include cobalamin malabsorption, congenital transport disorders, or a B12 poor diet [35]. Additional supplementation of B12 is needed due to increased demand for methyl donor SAM to support large epigenetic changes such as histone and DNA methylation during tissue regeneration involving cell differentiation [36].

High MS expression is typically observed in proliferating cells like epithelial cells and cancer cells [37]. Reactions dependent on THF derivatives are essential for synthesizing nitrogenous bases [38], so a deficiency in MS activity may lead to DNA damage, including mitochondrial DNA damage [39], due to nucleotide synthesis disruption, particularly of dTMP. Tumor-initiating cells are strongly dependent on methionine, and a deficiency of methionine in cancer cells caused by depletion of B12 and methionine or interventions in the methionine cycle inhibits cancer cell division [40,41].

#### 2.3.2. B12 as a Cofactor of Methylmalonyl-CoA Mutase

AdoCbl serves as a cofactor for the mitochondrial enzyme MMUT, which functions as a radical reservoir during catalysis. MMUT catalyzes the conversion of L-methylmalonyl-CoA to succinyl-CoA, an intermediate in the tricarboxylic acid (TCA) cycle. This enzymatic reaction is critical because D-methylmalonyl-CoA, but not L-methylmalonyl-CoA, can be enzymatically converted into methylmalonic acid (MMA), a tumorigenic metabolite [42]. Failure of MMUT catalysis will lead to accumulation of MMA and aberrant methylmalonylation disrupting cellular metabolism [43]. Spontaneous methylmalonylation affects the enzymes involved in key intracellular metabolic pathways, including sirtuin SIRT5, several enzymes in the TCA and urea cycles, and glutamate dehydrogenase 1 in glycolysis [43].

### 2.4. Non-Enzymatic Functions of B12

The non-enzymatic functions of B12 remain relatively unexplored. The presence of B12 in its free form is limited within cells, suggesting a tight regulation of its availability. Nevertheless, the unique structure of B12—comprising the corrin ring, nucleotide, and aryl hydrocarbon moiety—implies that each component may have distinct functional roles.

MeCbl, but not AdoCbl, is capable of binding to gasdermin E [44]. Gasdermins are key mediators of pyroptosis, a form of programmed cell death implicated in diseases and pathological processes [45]. Gasdermin E is expressed ubiquitously and can be cleaved and activated by various caspases following chemotherapy or infections, as well as by granzyme B released from cytotoxic lymphocytes. The binding of MeCbl to gasdermin E occupies Cys180, a critical residue for caspase and granzyme B activity, thereby preventing gasdermin E cleavage and subsequent pyroptosis. Supplementation with MeCbl protected mice from liver failure, highlighting its potential therapeutic significance.

B12 has the ability to directly bind to the aryl hydrocarbon receptor (AhR), functioning as an antagonist [46] by specifically interacting with the aryl moiety (base, see Figure 1A) and competing with AhR agonists such as TCDD (2,3,7,8-tetrachlorodibenzo-p-dioxin), which inhibits their effects. The binding of B12, along with folic acid (FA), prevents the nuclear localization of AhR, thereby diminishing transcriptional activation triggered by its agonists. Treatment with B12, FA, or their respective aryl moieties can reverse phenotypes associated with nutritional deficiencies, as seen in mice administered B12, FA, or their aryl derivatives, which showed protection against TCDD-induced conditions including anemia, thrombocytopenia, hepatic steatosis, and congenital anomalies like cleft palate. Notably, mice on a B12- or FA-deficient diet displayed symptoms similar to those on a control diet supplemented with low doses of TCDD, while AhR-null mice did not exhibit these symptoms even when on an FA-deficient diet, suggesting that some manifestations of B12 and FA deficiency are mediated by the over-activation of AhR.

## 3. B12 Deficiency

### 3.1. B12 Deficiency Causes

B12 deficiency can arise mainly from malabsorption (e.g., due to autoimmune conditions), defects in cellular uptake and delivery, insufficient dietary intake (like in the case of vegetarian or vegan diets [47]), toxins (e.g., exposure to nitric oxide) [48], and insufficient production of intrinsic factors [49]. Although B12 deficiency is more commonly observed in older adults due to the higher prevalence of malabsorptive conditions [50], it affects individuals across all age groups, particularly in regions where food insecurity is prevalent. The most frequent cause of B12 malabsorption is autoimmune gastritis (AIG), which causes deprivation of IF and gastric acid [51], while other causes are summarized in Table 1. Depending on deficiency etiology, various routes of B12 administration are utilized, such as oral and parenteral, topical, or intramuscular administration [52]. B12 insufficiency deficiency relates to conditions when level of B12 in the blood is within the standard (200 to 600 pmol/L) but biomarkers of B12 deficiency, such as elevated Hcy and MMA, are present.

Mutations in proteins involved in B12 processing lead to B12 deficiency. These proteins were found through genetic screens of patients with functional B12 deficiency. Historically, these proteins were called mut and CblN, where N designates a letter from A to G, J, or X [53,54]. Mutations in these proteins lead to various phenotypes (Table 2) characterized by methylmalonic acidemia, homocystinuria, or a combination of them from infancy and sometimes in adulthood. Methylmalonic acidemia is characterized by increased concentration of methylmalonic acid in body fluids, and the symptoms include (but not limited to) vomiting, weight loss, hypoglycaemia, neurological deterioration with hypotonia, irritability, lethargy, and the inability to thrive. The biochemical markers include metabolic acidosis, ketosis, hyperlactataemia, and hyperammonaemia [55].

These proteins include not only proteins that are directly interact with B12 but also proteins which ensure proper localization of such proteins. For example, endosomal ABCD4 transfers B12 from lysosome to the cytosol: mutations in LMBD1 protein, which directs ABCD4 to lysosomes, leads to B12 deficiency with elevated Hcy and MMA [56].

In humans, mutations in the TCN2 and SLC46A1 genes, which encode TC and a folate transporter, respectively, result in elevated levels of AhR (Aryl hydrocarbon Receptor) transcriptional activation [46]. In contrast, mutations in the MTHFR and MTR genes, responsible for encoding methylenetetrahydrofolate reductase and methionine synthase, do not influence AhR transcriptional activity. This suggests that at least some symptoms associated with vitamin B12 deficiency—such as anemia, fatty liver, and birth defects—are primarily driven by AhR overactivation rather than defects in one-carbon metabolism [46].

### 3.2. B12 Associated Conditions

The severity and consequences of B12 deficiency vary depending on its extent and duration, while the complex machinery of B12 transport and processing is susceptible to dysfunction at various stages. Inherited diseases are rare, mostly diagnosed in early infancy, and share common features like delayed development and inability to thrive; however, functionally mild mutations in B12-processing proteins may have consequences in adulthood. Consequences of severe B12 deficiency are mainly hematological (i.e., pernicious anemia) and neurological (i.e., neuron demyelination) [57]. B12 is involved in inflammation (reviewed elsewhere [4]).

Disrupted dTMP and DNA synthesis due to severe B12 and/or folate deficiency primarily affects cells with rapid turnover, such as in hematological system [58,59]. A lack of B12 arrests cell maturation caused by an elongation of the S phase of cell cycle [60]. This is caused by failed conversion of deoxyuridine to deoxythymidine and the subsequent dUTP incorporation into DNA instead of dTTP. In bone marrow, this results in larger than normal red cells and granulocyte precursors, among other morphological changes. Such impaired megaloblastic hematopoiesis affects all blood cells but erythrocytes are the most affected [58]. Symptoms of such anemias include weakness, lightheadedness, and shortage of the breath [58]. A lemon-yellow color of skin is developed due to hemolysis.

B12 deficiency is linked to diabetic neuropathy, where supplementation improves symptoms, particularly in patients on metformin, which can worsen B12 deficiency [61,62]. It alleviates symptoms of postherpetic and trigeminal neuralgia [63,64]. In the central nervous system, B12 deficiency disrupts myelination by increasing myelinotoxic cytokines and reducing myelinotrophic factors [65]. Supplementation is beneficial in conditions like multiple sclerosis, degenerative cervical myelopathy, and hepatic encephalopathy [66,67,68].

B12 is essential for one-carbon metabolism, and its deficiency can impair SAM-dependent monoamine biosynthesis, increasing the risk of mental disorders [69,70]. Hypomethylation of the monoamine oxidase A gene may be linked to B12 deficiency and it correlates with panic disorder and post-traumatic stress disorder [71,72]. B12 deficiency is associated with depressive symptoms, autism, and schizophrenia, often due to elevated homocysteine and impaired MS activity [73,74]. Supplementation can alleviate hallucinations and catatonia in psychiatric patients [75,76].

## 4. B12 Deficiency Treatments

### 4.1. Traditional Formulations

Current clinically approved approaches for B12 deficiency treatment include intramuscular injections, oral administration and nasal sprays [77]. Since injections may be painful and require qualified personnel, oral administration has become a possible alternative. Thus, Eligen^®^ technology was successfully applied and demonstrated efficiency comparable with intramuscular injections [78]. Nasal delivery of B12 became another alternative released to the market [79].

B12 is notable for having the lowest recommended daily allowance [80] and an intricate absorption and assimilation process, as highlighted in Section 2. Nonetheless, it is worth highlighting that being extremely hydrophilic, B12 necessitates constant administration and bioavailability, and for this reason, the development of B12 delivery strategies is extremely relevant [81]. In this scenario, typical pharmaceutical formulations, such as emulsions and tablets based on natural/approved products, are thoroughly investigated but are not the main focus of this work. However, notable advances in the field are continuously investigated and developed. For example, Karbalaei-Saleh et al. [82] optimized sunflower oil-based nanoemulsions for improving the oral delivery of B12. The researchers developed spontaneous emulsification and refined the concentrations of sunflower oil, Tween 80 surfactant, and vitamin B12 to create emulsions with maximum viscosity and entrapment efficiency as well as minimum particle size, polydispersity index, and turbidity. Without the use of high-energy treatment, these emulsions achieved an encapsulation efficiency of approximately 50% and a particle size <400 nm. A mathematical model for the prediction of the emulsions properties was also developed. Like for many other drugs, also for B12, oral administration represents the ideal administration routes and different efforts developed in this direction. In this scenario, B12 has been formulated into mucoadhesive tablets [83] as an alternative to intramuscular injections. It was shown that hydroxypropyl methyl cellulose/Carbopol 971P-based tablets placed in the buccal cavity of rabbits provided 2.7-fold better bioavailability of B12 compared to intramuscular injections.

#### 4.1.1. Use of Colloidal Systems for Delivering B12

Encapsulating B12 is very important because of the sensitivity of this vitamin to light, heat, and acidic conditions. To further increase B12 prolonged release and bioavailability, different encapsulation strategies based on colloidal systems and nanoformulations have been developed. Nanoemulsions, nanoparticles, liposomes, and polymeric carriers and fibers [84] have been optimized to improve B12 bioavailability, stability, and targeted delivery, addressing limitations associated with B12’s hydrophilic nature and large molecular size. For example, PLGA-based nanoparticles synthesized by Kuznetsova et al. [85] were coated with poly(ethylenimine) for enhancing biological barriers’ crossing and with dextran sulfate for improving their biocompatibility. In this case, B12 was successfully incorporated into the polyelectrolyte coating of the nanoparticles, while the PLGA core allowed for the loading of hydrophobic compounds. The authors also demonstrated a slow release rate of B12 under normal environmental conditions, enabling the storage of the prepared nanoparticles in suspension. Additionally, there was an increased release at the physiological temperature due to the interactions between B12 and dextran sulfate. Encapsulation of B12 was commonly tested also in multivitamin formulations. For achieving simultaneous delivery of B6 and B12, Karoshi et al. [86] encapsulated these vitamins in zein/gum arabic nanocarriers. These carriers demonstrated high stability over 90 days and provided improved uptake of vitamins in Caco-2 cells and increased bioavailability in rats. Encapsulation efficiency of B12 in this material was found >50% for the majority of formulations. Spray-drying is currently considered one of the best techniques to encapsulate sensitive molecules like vitamins, sometimes enhancing their efficacy [87]. Bajaj et al. [87] optimized the spray drying process for co-encapsulating B12 and D3 vitamins in gum acacia/modified starch/maltodextrin particles. While the encapsulation efficiency for B12 was around 70%, its bioavailability was 150% higher compared to oral administration. The fabrication of B12-loaded zein microparticles and fibers, by using electrospray/electrospinning or spray drying method, was optimized by Coelho et al. [88], reaching an encapsulation efficiency of 71–95%. B12 encapsulated in nanovesicles such as liposomes and niosomes can be eventually lyophilized using maltodextrin as a cryoprotectant. This process helped preserve the structural integrity of the vesicles and the stability of the loaded B12 during storage and transportation. In this scenario, Marchianò et al. [89] synthesized B12-loaded vesicles (Phospholipon 90G- or Span 60/cholesterol-based) and demonstrated their long-term stability under storage thanks to their lyophilization. Lyophilized nanovesicles can be used in various fields, including pharmaceuticals, cosmetics, and food industries, due to their ability to carry both hydrophilic and lipophilic compounds.

#### 4.1.2. Colloidal Systems Designed for B12 Delivery in the Nervous System

B12 is crucial for maintaining the health of the nervous system, and its deficiency can lead to various neurological and cognitive disorders. Symptoms of B12 deficiency include apathy, agitation, irritability, growth retardation, and delayed myelination or demyelination of nerve fibers [90,91,92,93]. Severe consequences may include funicular myelosis, sensorimotor polyneuropathy, optic neuropathy, and cognitive impairments [94]. Neuronal damage is often linked to hyperhomocysteinemia and impaired MS activity, which can be mitigated by hydroxocobalamin, betaine, and folate administration [95]. Hyperhomocysteinemia exacerbates neuronal apoptosis and oxidative stress, which can be reduced by NMDA and mGluR1 receptor antagonists [96]. Animal studies showed that B12 supplementation reduced inflammation and apoptosis while restoring neurotrophic and synaptic plasticity factors [97,98]. Maternal B12 deficiency during gestation can impair offspring learning and memory due to increased apoptosis and hyperhomocysteinemia [99]. Supplementing B12 in the elderly is beneficial for delaying cognitive decline, especially in those with high Hcy levels, as shown by clinical trials and meta-analyses [47].

Given its beneficial effects in the central nervous system, proper encapsulation designs were developed to improve the ability of B12 to overcome the blood–brain barrier. For example, Andrade et al. [100] prepared transferrin-functionalized liposomes loaded with B12. Transferrin represents probably the most investigated surface functionalization to enhance the passage through the brain–blood barrier and the targeting to neuronal cells. In this case, the liposomes were <200 nm in size and demonstrated stability under storage conditions for up to 2 months. Similarly, thanks to the ability of polymer nanoparticles to penetrate blood–brain barrier, Guler et al. [101] synthesized B12-loaded chitosan/tripolyphosphate/polyvinyl alcohol nanoparticles embedded in polyvinylpyrrolidone (PVP) and polyvinylpyrrolidone/polycaprolactone (PVP/PCL) nanofibers for sublingual and transdermal applications via pressurized gyration approach. Encapsulation efficiency was found around 60–80% for the developed system. The PVP fibers showed extreme biocompatibility with a complete dissolution occurring within 4 s, while PVP/PCL nanofibers provided sustained drug release over 12 days. Additionally, both types of nanofibers showed a notable anti-Alzheimer effect on Aβ1-42-induced SH-SY5Y cells with no significant cytotoxic effects observed.

#### 4.1.3. Colloidal Systems Designed to Deliver B12 for Other Conditions

B12 supplementation in autoimmune rheumatic diseases alleviates symptoms, albeit most likely in patients is B12 insufficiency rather than B12 deficiency (reviewed in [102]. In the mice model of arthritis, hydroxycobalamin, a form of B12, ameliorated the severity of arthritis and reduced citrullination, a common consequence of arthritis, due to inhibition of the citrullination mediator, peptidylarginine deiminase isoform 4 [102]. Belal et al. [103] proposed inorganic nanoparticles (zeolites and hydroxyapatite) as B12 carriers for arthritis treatment [104]. This technology demonstrated anti-inflammatory and antioxidant activities while reducing TGF-β mRNA expression in the Complete Freund’s adjuvant-induced arthritis rat model.

Associations between vitamin B12 and several pathological skin conditions such as vitiligo, atopic dermatitis, and aphthous stomatitis were found [105]. Topical formulations of B12 were proposed to treat skin conditions. B12 administration in the form of cream demonstrated significant efficacy in preventing or ameliorating atopic dermatitis, eczema, and psoriasis [106].

While passive diffusion through the skin is considered a safe and non-invasive administration route, it can be achieved only with molecules characterized by low polarity, an intermediate partition coefficient, and a small molecular size (less than 500 Da) [107]. Unfortunately, B12, with a molecular weight of 1355 Da, does not meet these criteria, making it poorly suited for passive diffusion; for this reason, this molecule needs particular drug delivery formulation. To overcome this limitation, Guillot et al. [108] prepared Phospholipon 90G-based vesicles with a maximum encapsulation efficiency of about 35%. Further in vitro experiment demonstrated that B12 absorption could be improved by pre-treatment of skin with microneedle arrays [109] to maximize B12 permeability in the stratum corneum [110], the most superficial layer of the skin. Poly(vinyl alcohol)/poly(vinylpirrolidone) dissolving microneedle B12-loaded arrays were effective in decreasing the inflammation in murine model.

B12 is also involved in wound healing via the production of SAM for epigenetic reprogramming of precursor cells and the reduction in homocysteine levels in plasma [36]. For this reason, Farzanfar et al. fabricated electrospun poly(caprolactone)/gelatin fibrous scaffolds containing B12 as potential wound-healing materials [111]. B12-loaded scaffolds provided >90% wound closure in 14 days in the Wistar rat model. Bhattacharyya et al. [112] engineered gelatin-based microspheres incorporating carbon dots and metal–organic frameworks (MOFs). The synergistic action of Zn^2+^ ions, imidazole groups, and carbon dots endowed the microspheres with potent antibacterial activity against both Gram-negative and Gram-positive bacteria. Additionally, integrating vitamin B12 into the MOFs facilitated its rapid release in an acidic environment. In vitro wound healing assays demonstrated that these synthesized microspheres significantly enhanced fibroblast proliferation, highlighting their potential for therapeutic applications.

**Figure 3:**
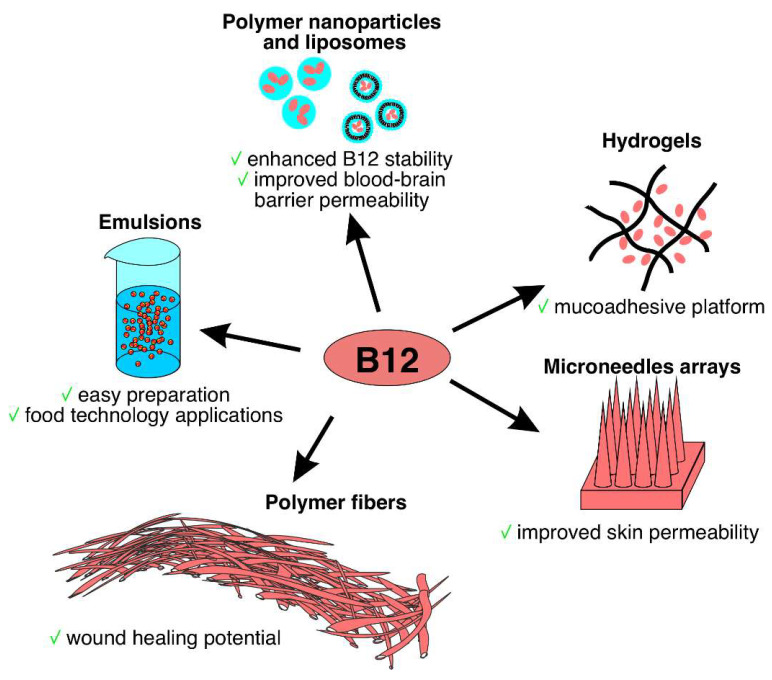
Different formulations of B12 delivery systems.

It was demonstrated that B12 can reduce oxidative stress and ocular surface disease index [113], enhancing corneal re-epithelization and re-innervation [114] at the same time. Thus, B12 became a promising component of eye drops for dry eye disease (DED) treatment. Yang et al. proposed nebulized B12 saline solution for the treatment of DED [115]. In vivo experiments demonstrated improved epithelial cell density and increased tear break-up time for the group treated with B12. However, even commercially available eye drops containing B12 demonstrate short protective effects [116]. Mohamad et al. proposed a Pluronic-based hydrogel as a long-term action platform [117]. The authors demonstrated B12 release for up to 12 days, which resulted in an improved tear flow. In conclusion, the exploration of different B12 delivery strategies had and has significantly advanced its therapeutic potential for ameliorating various diseases. The colloidal systems utilized for B12 delivery as well as their applications and advantages are summarized in Table 3. Figure 3 summarizes the major benefits of encapsulating B12 and some of their potential applications.

Recent studies have highlighted potential risks associated with vitamin B12 overdose. High doses of cyanocobalamin can lead to various adverse effects, including anxiety and insomnia [118]. In rare cases, B12 overdose may trigger neurological (i.e., bipolarism) [119] and skin disorders like acne exacerbation and rosacea fulminant [120]. Additionally, excessive vitamin B12 supplementation can modify gut microbiota, potentially increasing susceptibility to pathogens. In mice, elevated B12 intake facilitated Citrobacter rodentium colonization and pathogenesis by impacting Lachnospiraceae populations and reducing microbial diversity [121]. These findings suggest that while B12 supplementation is generally considered safe, excessive intake may have unexpected consequences on human health and microbial ecology, emphasizing the importance of appropriate dosing and monitoring.

## 5. B12 to Improve Drug Delivery in Pathological Conditions: Focus on Cancer and Other Diseases

Vitamin B12 showed interesting properties in the treatment of cancer due to its unique ability to target rapidly proliferating cells. On the other hand, its effective absorption and favorable physicochemical characteristics support the development of pharmaceutical formulation designed for enteral administration. Vitamin B12 is water-soluble (10 mg/mL), which is attributed to the presence of several polar functional groups [79]. It partitions mainly into aqueous phases rather than organic solvents [122]. B12 is most stable under neutral to slightly alkaline pH conditions at low temperatures, with a half-life ranging from 100 to 250 days. In contrast, at pH 2 and 37 °C, its half-life is approximately 8 days [122]. These characteristics enable B12 to function as a powerful targeting ligand for developing novel drug delivery systems. By harnessing the natural uptake and trafficking mechanisms of B12, researchers are developing innovative technologies that minimize off-target effects and optimize therapeutic outcomes. Consequently, in this separate section, we have detailed B12’s impact on drug efficacy, safety, and pharmacokinetic properties with a particular focus on cancer, considering the great effort performed in this direction. The last section instead is dedicated to B12-based formulation of other drugs for the treatment of infectious diseases and diabetes.

### 5.1. B12-Targeted Nanoparticles for Cancer

As aforementioned B12 is an essential nutrient for rapidly growing tissues and, for this reason, its utilization in cancer therapy (i.e., in leukemia, lymphoma, melanoma, and some solid tumors, overexpressing the receptor of transcobalamin-CD320, making this vitamin an ideal targeting ligand [123]).

Gupta et al. [124] developed doxorubicin-loaded sterically stabilized liposomes conjugated with B12 for tumor targeting (SL-VB12). These liposomes demonstrated enhanced cellular uptake and cytotoxicity against B16F10 melanoma cells compared to non-targeted liposomes. SL-VB12 significantly increased tumor accumulation and prolonged survival in tumor-bearing mice, highlighting the potential of B12 as a targeting ligand in cancer therapy. Another study [125] highlighted the efficacy of B12-loaded solid lipid nanoparticles (B12-SLNs), which exhibited higher cytotoxicity against H-Ras SRP7 cancer cells compared to free B12, while showing minimal effects on NIH/3T3 fibroblasts. With an average size of 200 nm, these nanoparticles provided sustained drug release and high entrapment efficiency, making them suitable for controlled drug delivery.

A novel targeted drug delivery system combining B12 and cisplatin-loaded porous silica nanoparticles via coordination bonds was developed [126]. The system leveraged B12 as an active targeting unit to enhance its specificity for cancer cells, while the porous silica nanoparticles supported controlled drug release. B12 also represented a gatekeeper to prevent premature drug release, offering a promising approach to reduce side effects and enhance the efficacy of cisplatin in cancer therapy.

Functionalization of B12 onto diatom microalgae surface was used to create a biomaterial for targeted drug delivery, enhancing the affinity of the drug-loaded particles to colorectal (HT-29) and breast cancer (MCF-7) cell lines [127]. This formulation demonstrated an increased uptake and retention of the anticancer agent in tumor tissues, particularly in environments with high CD320 expression. The slow, targeted release of the ruthenium-based anticancer agent from the B12-functionalized diatoms showed promising results for improving the efficacy of cancer treatment while minimizing off-target effects.

Guo et al. [128] developed B12-conjugated sericin micelles loaded with paclitaxel for targeted therapy of CD320-overexpressed gastric cancer. The micelles demonstrate enhanced cellular uptake, improved drug delivery, and the ability to reverse drug resistance both in vitro and in vivo, while maintaining good biocompatibility. Additionally, B12-conjugated PLGA-PEG nanoparticles (PLGA-PEG-VB12 NPs) effectively delivered microRNAs-532-3p to CD320-overexpressing gastric cancer cells, significantly reducing the expression of the apoptosis repressor ARC. This led to mitochondrial damage, including loss of membrane potential, increased reactive oxygen species, and activation of the caspase-dependent apoptosis pathway, ultimately suppressing cancer cell proliferation both in vitro and in vivo.

**Figure 4:**
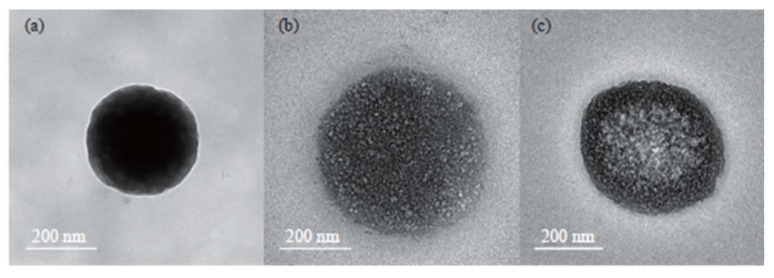
Different formulations of chitosan nanoparticles (NCV): Transmission electron microscopy (TEM) of (**a**) empty carriers, (**b**) vitamin (B12, C and D3)-loaded carriers, and (**c**) vitamins and cysplatin-loaded carriers. Published with permission from Othayoth et al., 2023 [129].

B12 has been incorporated into combination therapies to enhance the efficacy of anticancer drugs while mitigating chemotherapy-related fatigue (CRF). For instance, multivitamin (including vitamin B12, D3, and C) and cisplatin-loaded chitosan nanoparticles (NCV) revealed the reduction in CRF without compromising the anticancer properties of cisplatin [129] (Figure 4). NCV formulation modulated the expression of genes associated with cancer proliferation, such as DDX3X and Ki-67, in various cancer cell lines, suggesting a synergistic effect of the vitamins and cisplatin in combating cancer.

### 5.2. B12—Drug Conjugates

Conjugating B12 with cytotoxic drugs or drug-polymer complexes, such as daunomycin-pHPMA, has demonstrated enhanced tumor uptake and increased anticancer efficacy in various murine cancer models [130]. This targeted approach leverages the natural uptake mechanisms of B12, improving drug delivery to tumor cells while potentially reducing off-target toxicity. Ruiz-Sánchez et al. [131] conjugated B12 with platinum-based drugs to form B12-CN-PtII complexes, which act as prodrugs releasing cytotoxic PtII agents intracellularly after enzymatic reduction. These conjugates demonstrated selective uptake and cytotoxicity in cancer cells, particularly when bound to TCII, enhancing their therapeutic potential. Additionally, B12 derivative conjugated with a fluorophore (Bodipy650-cobalamin) demonstrated selective accumulation in pancreatic adenocarcinoma (PDAC) tumors, both in flank and orthotropic models, with minimal off-target effects. The cobalamin platform was shown to release its cargo upon activation by both near-infrared (NIR) light and X-ray radiation, making it a promising tool for spatiotemporally controlled drug delivery in combination with radiotherapy. Mehder et al. [132] developed fluorescent vitamin B12–platinum(II) derivatives to achieve tumor-targeted delivery of therapeutic agents. These metal conjugates were engineered in the form [FLUO–B12–M], where vitamin B12 was modified at the 5′-hydroxo group of the ribose with the fluorophore rhodamine 6G, a modification that did not interfere with the vitamin’s receptor interactions. The Co(III)–cyano group was linked to a metal-based structure M, which included a Pt(II) substrate with phenylamino-oxime ligands derived from either R- or S-limonene. These differences in ligand composition influenced the derivatives’ biological activity, lipophilicity, and interaction with tumor cells, ultimately affecting their efficacy as potential metallotheranostic agents. This approach followed a typical Trojan horse strategy, leveraging vitamin B12’s intracellular transformation into its active forms, methylcobalamin and adenosylcobalamin, through the reduction in Co(III) to Co(II) and the subsequent release of the cyano group. This design facilitated preferential accumulation of the conjugates in cancer cells, resulting in the intracellular release of the cytotoxic payload. Additionally, rhodamine 6G enabled tracking and evaluation of therapeutic transport and distribution through fluorescence spectroscopy. Confocal microscopy confirmed their internalization into cancer cells via energy-dependent endocytosis, and preliminary evaluations indicated moderate binding affinities to DNA, further supporting effective targeting. The dual functionality of vitamin B12 as both a therapeutic carrier and imaging agent positions these derivatives as promising candidates for cancer treatment and monitoring.

### 5.3. B12 for Cancer Imaging

In modern medicine, vitamins are increasingly utilized in molecular imaging techniques like Single Photon Emission Computed Tomography (SPECT) and Positron Emission Tomography (PET) for cancer detection and monitoring [133]. These noninvasive methods can rely on radiolabeled vitamin analogs to target tumor cells, enabling precise localization and staging of malignancies [134,135]. Radiolabeled vitamins offer advantages such as rapid clearance from blood and low immunogenicity, making them promising candidates for imaging and therapeutic applications [136]. Transport mechanisms, including the overexpression of specific carriers and receptors, facilitate vitamin delivery to malignant tissues, enhancing tumor specificity [137]. Recent advancements focus on optimizing these radiopharmaceuticals for improved diagnostic accuracy and therapeutic outcomes, although challenges like off-target uptake and radiation exposure remain [138]. Overall, integrating vitamins into nuclear medicine exemplifies their role as innovative tools in combating diseases like cancer. As mentioned before, B12 can bind to two main transport proteins, including HC in the saliva and TC in the plasma, and onto cancer cell surface. B12-based imaging technologies have been investigated in the last 30 years, and different studies have shown strong binding to receptors like megalin in the kidneys and significant tumor uptake with radiolabeled forms, such as [111In]In-DAC and [99mTc]Tc-DTPA-b-cyanocobalamin. Clinical trials indicate that these radiotracers can effectively differentiate malignant tissues from normal ones, particularly in breast cancer, suggesting further investigation into their targeting capabilities is warranted. These efforts paved the way for clinical application, including cancer screening based on tumor metabolic features like in the case of breast cancer. In a recent study, In111-labeled B12 was utilized to detect breast tumors prior to surgery in both human patients and mice. The radiolabeled B12 analog, [111In]AC, showed significantly higher uptake in 18 tumors compared to normal breast tissue [133]. Aggressive triple-negative (TN) and HER2-positive tumors exhibited greater tumor-to-background ratios than estrogen receptor (ER) and progesterone receptor (PR)-positive tumors. Metastatic sites had higher ratios than primary tumors, particularly in patients with elevated serum B12 levels, likely due to recent B12 or dexamethasone intake. The study indicated that chronic B12 intake might contribute to higher tumor/background ratios, as elevated B12 concentrations could reduce CD230 levels in normal tissues while increasing them in tumors. Dexamethasone may have a similar effect by raising circulating B12 levels. Tumor accumulation of B12 might occur via alternative pathways, including megalin and cubam receptors and haptocorrin, which is overexpressed in some tumors. Elevated levels of HC and TC receptors have been observed in 50–65% of breast cancer patients, suggesting potential biomarkers. The findings recommend that clinical imaging of B12 uptake in breast tumors should involve administering the vitamin before injecting the radiolabeled analog to improve tumor detection sensitivity.

### 5.4. B12 as Anticancer Adjunctive Therapy

**Figure 5:**
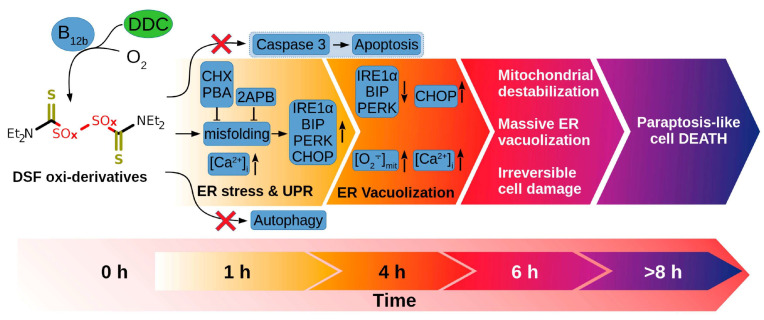
Initiation of paraptosis in cancer cells mediated by the combination of B12 and DDC. Published from Solovieva et al. [140].

B12 can also be used to enhance the efficacy of different therapeutics directly or indirectly by enhancing their pharmacokinetics properties. For example, it was recently shown [139] that while B12 has mild effects on cancer cell proliferation, it can increase the toxicity of the active form of vitamin D3 (1,25(OH)_2_D_3_) on different tumor cell lines including HeLa, HL-60, and MCF-7, without affecting normal cells (MCF10A). The mechanism standing behind this synergistic activity was primarily apoptosis caspase 4- and 8-dependent, while exhibiting no release of cytochrome c from mitochondria, suggesting a unique apoptotic pathway. Additionally, treated cells show reduced adhesion and altered morphology, indicating disorganized actin cytoskeleton and decreased cell polarization. This research is important in the field because it highlights the potential role of vitamin B12 in enhancing the efficacy of established anticancer agents as an adjunctive treatment option for certain cancer types. A similar effect was demonstrated also by Solovieva et al. [140], who demonstrated that B12 can synergistically enhance the anticancer effects of diethyldithiocarbamate (DDC) against HEp-2 cells. In their work, the authors highlighted the mechanisms underlying this interaction, demonstrating that B12b enhanced the toxicity of DDC by catalyzing the formation of reactive disulfiram (DSF) derivatives, leading to a unique cell death pathway distinct from apoptosis, autophagy, or necrosis. The study revealed that this combination induced severe endoplasmic reticulum (ER) stress, nuclear deformation, extensive vacuolization, and an increase in intracellular calcium levels, which ultimately resulted in paraptosis-like cell death (Figure 5). The findings emphasize the potential of vitamin B12b as a critical agent in enhancing the cytotoxicity of DDC, suggesting its role in developing targeted cancer therapies.

B12 is necessary to sustain cellular high proliferation, and its deficiency is common in cancer patients, often due to malabsorption or the cancer itself affecting the gastrointestinal tract or bone marrow. Deficiency can cause hematological and neurological problems that require treatment [141]. Epidemiological studies provide mixed results on the association between dietary or supplemental vitamin B12 intake and cancer risk: while elevated plasma B12 levels have been associated with increased solid cancer risk, particularly in metastatic cases [142], the causal link between high B12 intake or plasma levels and cancer remains uncertain [141].

Some studies indicate that high dietary or supplemental intake of B12, especially in men and smokers, is associated with an increased risk of lung cancer [143]. The relationship may depend on factors such as smoking status, sex, genetic background, and baseline B12 levels, making it difficult to draw definitive conclusions. Overall, while elevated B12 levels may serve as a potential cancer marker, more research is needed to establish causality and determine optimal supplementation practices.

### 5.5. B12 Formulation to Improve Enteral Delivery of Antibiotics

Vitamin B12 can play a crucial role in enhancing the intestinal permeability of antimicrobial conjugates by acting as a targeting ligand that facilitates effective absorption in the small intestine [144]. It has to be considered also that B12 has anti-inflammatory properties. For example, along with other B vitamins, B12 showed immunomodulatory, anti-inflammatory, and antioxidant properties in sepsis management [145].

In this context, a novel antibiotic formulation was developed by synthesizing conjugates of the peptide antibiotic colistin (CT) with the biocompatible glycopolymer poly(2-deoxy-2-methacrylamido-D-glucose) (PMAG), incorporating both deferoxamine (DFOA) and vitamin B12 to improve bioavailability and reduce cytotoxicity. A complex drug formulation for CT is recommended to mitigate potential side effects to the kidneys and the nervous system as well as to improve its enteral absorption. They confirmed the structures of different conjugates (with or without B12) through various physicochemical methods and demonstrated that vitamin B12 significantly increased the permeability of the CT conjugates across the Caco-2 cell monolayer. Biological evaluations revealed that all conjugates exhibited lower cytotoxicity compared to free CT, with antimicrobial efficacy varying based on conjugate composition; those linked by hydrolyzable bonds showed enhanced activity, while conjugates containing DFOA complexed with Fe^3+^ exhibited superior antimicrobial activity against Pseudomonas aeruginosa. The authors concluded that their PMAG-based conjugates represent a promising strategy for targeted antibiotic delivery, addressing critical challenges in antibiotic resistance and toxicity. A similar design and effect were proposed by generating hyaluronic acid nanoparticles (size of 100 nm) loaded with CT and modified with vitamin B12 (Succinyl cyanocobalamin), further confirming the role of this vitamin in improving oral delivery [146]. Singh et al. [147] exploited the ability of B12 to be absorbed in by the gastrointestinal tract to develop a new nanoformulation based on solid lipid nanoparticles to deliver amphotericin B and improve current management of leishmaniosis. In this case, vitamin B12 was used to decorate the particles.

Similarly, Sahadi et al. [148] highlighted the potential of B12 in improving the oral administration of insulin. In particular, the authors encapsulated this hormone in pegylated liposomes and functionalized their surface with vitamin B12. This technology was efficient in vitro and in vivo to protect the biological payload while efficiently increasing the passage through the intestinal tract and increasing the bioavailability of insulin. More importantly, the system did not show any sign of toxicity both in vitro and in vivo. Maiorova et al. [149] developed and characterized nanoparticles generated from a nucleotide-free analog of vitamin B12, specifically heptamethyl cobyrinate (ACCby), encapsulated in bovine serum albumin (BSA) nanocarriers. These nanoparticles exhibited significant neuroprotective effects in vivo, particularly in a rat model of seizures induced by thiosemicarbazide. The encapsulation enhanced the stability and bioavailability of ACCby, allowing it to maintain its neuroprotective properties without significant loss when administered in the encapsulated form. The study demonstrated that BSA nanocarriers effectively retained the nucleotide-free vitamin B12 analog, facilitating targeted delivery and improving therapeutic outcomes, as evidenced by the survival rates of treated rats compared to controls. Given the importance of BSA nanoparticles in the field of nanomedicine [150], this paper highlights an easy and cost-effective approach to deliver B12.

## 6. Conclusions

In conclusion, vitamin B12 serves not only as a vital nutrient but also as a promising candidate for enhancing drug delivery systems. The complex mechanisms of B12 absorption and metabolism reveal significant opportunities for improving the oral bioavailability of various therapeutics. Elderly patients with impaired gastrointestinal absorption would especially benefit from nanoemulsions, nanoparticles, or microcapsules designed to enhance oral B12 bioavailability once these formulations become commercially available. Given the high prevalence of B12 deficiency and its associated health risks, further research into B12-based delivery systems, especially their effectiveness on the organismal level, is warranted. Future studies should focus on in vivo studies in rodents or clinical trials to validate the efficacy of formulations tested in cellular models and optimization of B12 formulations’ administration protocols, thereby maximizing their therapeutic potential, particularly within the context of nanomedicine. By leveraging the unique properties of B12, researchers can develop innovative strategies to address challenges in drug delivery and improve patient outcomes, particularly in the advancement of cancer therapy. The overexpression of TCII-R in cancer cells presents an opportunity to enhance targeting through B12-conjugated drugs and nanoparticles, which can improve the specificity and efficacy of therapeutic agents while minimizing systemic toxicity. Research using models more complex than single-cell-line cultures is required, at least co-culture of healthy and tumor cells, to estimate off-target effects of B12-drug conjugates for cancer treatment. These innovations hold promise for enhancing the delivery of chemotherapeutic agents and other therapeutic molecules, paving the way for more targeted and personalized cancer treatments.

## Figures and Tables

**Table 1:** Causes of B12 deficiency. Reproduced with permission from [51].

Cause	Mechanism	Diagnosis
Intrinsic factor deficiency	Pathogenic mutations in CBLGIF	No AIG, family context Gene sequencing
Imerslund–Gräsbeck disease	Pathogenic mutations in CUBN and/or AMN	No AIG, family context Gene sequencing
Biermer–Addison’s disease	AIG with impaired IF and gastric acid secretion	Endoscopy with fundic and antral biopsies IF and parietal cell autoimmunity
Non–Biermer–Addison’s gastritis	Impaired gastric acid secretion Malabsorption of protein bound VB12	No AIG
Bariatric surgery and gastrectomies	Impaired IF and gastric acid secretion Bacterial overgrowth	
Pancreatic insufficiency	Impaired degradation of HC	Pancreas imaging, lipasemia, steatorrhea
Obstructive Jaundice	Impaired VB12 binding to IF and uptake	Imaging and biology
Tropical sprue and celiac disease	Villous atrophy with impaired VB12-IF uptake	Anti-tranglutaminase auto-immunity
Blind loop syndrome	Bacterial overgrowth	Breath test
Parasitic infestations	Villous atrophy and VB12 consumption	Stool analyses
Zollinger–Ellison syndrome	Acid intestinal pH and Impaired VB12-IF uptake	Imaging and gastrinemia
Inflammatory bowel diseases	Parietal injury with impaired IF-B12 uptake	Endoscopy, imaging
Chronic radiation enteritis of the distal ileum	Parietal injury with impaired IF-B12 uptake	Imaging
Iatrogenic effects of drugs	Impaired IF-B12 uptake	

**Table 2:** Defects in Cbl-processing proteins. Summarized from [53,54].

Defect in Protein	Protein Function	Phenotype
cblA (MMAA)	Loading of AdoCbl in MMUT and ejection of oxidized Cbl from MMUT	Methylmalonic acidemia, acidotic crises
CblB(MMAB)	Chaperone for MMAA and MMUT	Similar to CblA but more severe
CblC (MMACHC)	Decyanation, dealkylation of Cbl	Homocystinuria and methylmalonic acidemia, hematological, neurological and ophthalmic abnormalities
CblD (MMADHC)	Adapter for MMACHC for transfer of Cbl to MS or MMAA	Isolated methylmalonic acidemia or homocystinuria, or combined methylmalonic acidemia and homocystinuria
CblE (methionine synthase reductase)	Reduction in oxidized Cbl in MS	Similar to CblG
cblF (LMBD1)	Escort of ABCD4 to lysosomes with endocytosed VB12-TC	Mild methylmalonic acidemia and homocystinuria, developmental delay
CblG (MS)	Methylation of homocysteine to form methionine using MeCbl as the cofactor	Highly variable
CblJ (ABCD4)	Transport of free Cbl from lysosomes to the cytosol	Feeding difficulties, macrocytic anemia, and congenital heart defects
CblX (HCFC1)	Transcription coregulator HCFC1 controlling expression of MMACHC	Similar to CblC, milder metabolic representation but more severe developmental delay, choreoathetosis, and intractable epilepsy
mut	L-methylmalonyl-CoA conversion	Isolated methylmalonic acidemia

**Table 3:** Colloidal formulations used for the delivery of B12.

Formulation	Application	Particle Size, nm	Advantages	Reference
Nanoemulsion	Food enrichment with B12	329 ± 13	Simple, low-energy fabrication process	[82]
Nanoparticles	B12 delivery to bone marrow and nerve cells	207 ± 100	Stable aqueous dispersion, which does not require lyophiliation	[85]
	Food enrichment with B12	~170	Co-encapsulation of B12 and B6	[86]
	Food enrichment with B12	260–1400	Co-encapsulation of B12 and D3	[87]
Microcapsules	Food and nutraceutical applications	~10,000–15,000	Fabrication of microcapsules with specific morphology and controlled release	[88]
Liposomes	Cosmetics, food, and pharmaceutical applications	~30–100	N/A	[89]
	Alzheimer’s disease therapy	<200	Improved blood–brain barrier permeation	[100]
Niosomes	Cosmetics, food, and pharmaceutical applications	57 ± 18	Lower cost and easier functionalization compared to liposomes	[89]
Inorganic nanoparticles	Arthritis treatment	420–640	High loading capacity	[104]
	Arthritis treatment	~310	High loading capacity	[104]

## Abbreviations

The following abbreviations are used in this manuscript:

HC	Haptocorrin
TC	Transcobalamin
Cbl	Cobalamin
methylTHF	Methyltetrahydrofolate
THF	Tetrahydrofolate
MeCbl	Methylcobalamin
AdoCbl	Adenosylcobalamin
Hcy	Homocysteine
SAM	S-adenosylmethionine
SAH	S-adenosylhomocysteine
MS	Methionine syntase
MMUT	Mitochondrial methylmalonyl-CoA mutase
CD320	Receptor of transcobalamin complex with B12
MMA	Methylmalonic acid
TCA cycle	Tricarboxylic acid cycle
AhR	Aryl hydrocarbon receptor
HPLC	High-performance liquid chromatography
CT	Colistin

## References

[1] Mucha P., Kus F., Cysewski D., Smolenski R.T., Tomczyk M. (2024). Vitamin B12 metabolism: A network of multi-protein mediated processes. Int. J. Mol. Sci..

[2] Martens J.-H., Barg H., Warren M.a., Jahn D. (2002). Microbial production of vitamin B 12. Appl. Microbiol. Biotechnol..

[3] Degnan P.H., Taga M.E., Goodman A.L. (2014). Vitamin B12 as a modulator of gut microbial ecology. Cell Metab..

[4] Simonenko S.Y., Bogdanova D.A., Kuldyushev N.A. (2024). Emerging roles of vitamin B12 in aging and inflammation. Int. J. Mol. Sci..

[5] Blakeley M., Sobczyńska-Malefora A., Carpenter G. (2020). The origins of salivary vitamin a, vitamin B12 and vitamin D-binding proteins. Nutrients.

[6] Fyfe J.C., Madsen M., Højrup P., Christensen E.I., Tanner S.M., De La Chapelle A., He Q., Moestrup S.K. (2004). The functional cobalamin (vitamin B12)–intrinsic factor receptor is a novel complex of cubilin and amnionless. Blood.

[7] Kurpad A.V., Pasanna R.M., Hegde S.G., Patil M., Mukhopadhyay A., Sachdev H.S., Bhat K.G., Sivadas A., Devi S. (2023). Bioavailability and daily requirement of vitamin B12 in adult humans: An observational study of its colonic absorption and daily excretion as measured by [13C]-cyanocobalamin kinetics. Am. J. Clin. Nutr..

[8] Wexler A.G., Schofield W.B., Degnan P.H., Folta-Stogniew E., Barry N.A., Goodman A.L. (2018). Human gut Bacteroides capture vitamin B12 via cell surface-exposed lipoproteins. Elife.

[9] Juodeikis R., Jones E., Deery E., Beal D.M., Stentz R., Kräutler B., Carding S.R., Warren M.J. (2022). Nutrient smuggling: Commensal gut bacteria-derived extracellular vesicles scavenge vitamin B12 and related cobamides for microbe and host acquisition. J. Extracell. Biol..

[10] Brada N., Gordon M.M., Wen J., Alpers D.H. (2001). Transfer of cobalamin from intrinsic factor to transcobalamin II. J. Nutr. Biochem..

[11] Kitai K., Kawaguchi K., Tomohiro T., Morita M., So T., Imanaka T. (2021). The lysosomal protein ABCD4 can transport vitamin B12 across liposomal membranes in vitro. J. Biol. Chem..

[12] Beedholm-Ebsen R., van de Wetering K., Hardlei T., Nexø E., Borst P., Moestrup S.K. (2010). Identification of multidrug resistance protein 1 (MRP1/ABCC1) as a molecular gate for cellular export of cobalamin. Blood J. Am. Soc. Hematol..

[13] Cham N., Doyle R.P. (2025). Corrin Ring Modification in Peptide Drug Development—A Brief History of “Corrination”. ChemMedChem.

[14] Fedosov S.N., Fedosova N.U., Kräutler B., Nexø E., Petersen T.E. (2007). Mechanisms of discrimination between cobalamins and their natural analogues during their binding to the specific B12-transporting proteins. Biochemistry.

[15] Hardlei T.F., Nexo E. (2009). A new principle for measurement of cobalamin and corrinoids, used for studies of cobalamin analogs on serum haptocorrin. Clin. Chem..

[16] Quadros E.V., Nakayama Y., Sequeira J.M. (2009). The protein and the gene encoding the receptor for the cellular uptake of transcobalamin-bound cobalamin. Blood J. Am. Soc. Hematol..

[17] Gordon M., Howard T., Becich M., Alpers D. (1995). Cathepsin L mediates intracellular ileal digestion of gastric intrinsic factor. Am. J. Physiol.-Gastrointest. Liver Physiol..

[18] Deme J.C., Hancock M.A., Xia X., Shintre C.A., Plesa M., Kim J.C., Carpenter E.P., Rosenblatt D.S., Coulton J.W. (2014). Purification and interaction analyses of two human lysosomal vitamin B12 transporters: LMBD1 and ABCD4. Mol. Membr. Biol..

[19] Gherasim C., Hannibal L., Rajagopalan D., Jacobsen D.W., Banerjee R. (2013). The C-terminal domain of CblD interacts with CblC and influences intracellular cobalamin partitioning. Biochimie.

[20] Padovani D., Labunska T., Palfey B.A., Ballou D.P., Banerjee R. (2008). Adenosyltransferase tailors and delivers coenzyme B12. Nat. Chem. Biol..

[21] Takahashi-Iñiguez T., González-Noriega A., Michalak C., Flores M.E. (2017). Human MMAA induces the release of inactive cofactor and restores methylmalonyl-CoA mutase activity through their complex formation. Biochimie.

[22] Fedosov S.N., Nexo E., Heegaard C.W. (2024). Kinetics of Cellular Cobalamin Uptake and Conversion: Comparison of Aquo/Hydroxocobalamin to Cyanocobalamin. Nutrients.

[23] Boachie J., Adaikalakoteswari A., Goljan I., Samavat J., Cagampang F.R., Saravanan P. (2021). Intracellular and tissue levels of vitamin B12 in hepatocytes are modulated by CD320 receptor and TCN2 transporter. Int. J. Mol. Sci..

[24] Grasbeck R., Nyberg W., Reizenstein P. (1958). Biliary and Fecal Vit. B12 Excretion in Man. An Isotope Study. Proc. Soc. Exp. Biol. Med..

[25] Hsu J.M., Kawin B., Minor P., Mitchell J.A. (1966). Vitamin B12 concentrations in human tissues. Nature.

[26] Mascarenhas R., Gouda H., Ruetz M., Banerjee R. (2022). Human B12-dependent enzymes: Methionine synthase and Methylmalonyl-CoA mutase. Methods in Enzymology.

[27] Sanderson S.M., Gao X., Dai Z., Locasale J.W. (2019). Methionine metabolism in health and cancer: A nexus of diet and precision medicine. Nat. Rev. Cancer.

[28] Watkins D., Ru M., Hwang H.-Y., Kim C.D., Murray A., Philip N.S., Kim W., Legakis H., Wai T., Hilton J.F. (2002). Hyperhomocysteinemia due to methionine synthase deficiency, cblG: Structure of the MTR gene, genotype diversity, and recognition of a common mutation, P1173L. Am. J. Hum. Genet..

[29] Garcia M.M., Guéant-Rodriguez R.M., Pooya S., Brachet P., Alberto J.M., Jeannesson E., Maskali F., Gueguen N., Marie P.Y., Lacolley P. (2011). Methyl donor deficiency induces cardiomyopathy through altered methylation/acetylation of PGC-1α by PRMT1 and SIRT1. J. Pathol..

[30] Banerjee R.V., Matthews R.G. (1990). Cobalamin-dependent methionine synthase. FASEB J..

[31] Lu S.C. (1998). Methionine adenosyltransferase and liver disease: It’s all about SAM. Gastroenterology.

[32] Finkelstein J.D. (1990). Methionine metabolism in mammals. J. Nutr. Biochem..

[33] Li T., Chen Y., Li J., Yang X., Zhang H., Qin X., Hu Y., Mo Z. (2015). Serum homocysteine concentration is significantly associated with inflammatory/immune factors. PLoS ONE.

[34] Ding W., Smulan L.J., Hou N.S., Taubert S., Watts J.L., Walker A.K. (2015). s-Adenosylmethionine levels govern innate immunity through distinct methylation-dependent pathways. Cell Metab..

[35] Guéant J.-L., Guéant-Rodriguez R.-M., Kosgei V.J., Coelho D. (2022). Causes and consequences of impaired methionine synthase activity in acquired and inherited disorders of vitamin B12 metabolism. Crit. Rev. Biochem. Mol. Biol..

[36] Kovatcheva M., Melendez E., Chondronasiou D., Pietrocola F., Bernad R., Caballe A., Junza A., Capellades J., Holguín-Horcajo A., Prats N. (2023). Vitamin B12 is a limiting factor for induced cellular plasticity and tissue repair. Nat. Metab..

[37] Sullivan M.R., Darnell A.M., Reilly M.F., Kunchok T., Joesch-Cohen L., Rosenberg D., Ali A., Rees M.G., Roth J.A., Lewis C.A. (2021). Methionine synthase is essential for cancer cell proliferation in physiological folate environments. Nat. Metab..

[38] Baggott J.E., Tamura T. (2015). Folate-dependent purine nucleotide biosynthesis in humans. Adv. Nutr..

[39] Heyden K.E., Fiddler J.L., Xiu Y., Malysheva O.V., Handzlik M.K., Phinney W.N., Stiles L., Stabler S.P., Metallo C.M., Caudill M.A. (2023). Reduced methionine synthase expression results in uracil accumulation in mitochondrial DNA and impaired oxidative capacity. PNAS Nexus.

[40] Li F., Liu P., Mi W., Li L., Anderson N.M., Lesner N.P., Burrows M., Plesset J., Majer A., Wang G. (2024). Blocking methionine catabolism induces senescence and confers vulnerability to GSK3 inhibition in liver cancer. Nat. Cancer.

[41] Wang Z., Yip L.Y., Lee J.H.J., Wu Z., Chew H.Y., Chong P.K.W., Teo C.C., Ang H.Y.-K., Peh K.L.E., Yuan J. (2019). Methionine is a metabolic dependency of tumor-initiating cells. Nat. Med..

[42] Gomes A.P., Ilter D., Low V., Endress J.E., Fernández-García J., Rosenzweig A., Schild T., Broekaert D., Ahmed A., Planque M. (2020). Age-induced accumulation of methylmalonic acid promotes tumour progression. Nature.

[43] Head P.E., Myung S., Chen Y., Schneller J.L., Wang C., Duncan N., Hoffman P., Chang D., Gebremariam A., Gucek M. (2022). Aberrant methylmalonylation underlies methylmalonic acidemia and is attenuated by an engineered sirtuin. Sci. Transl. Med..

[44] Xu W., Wang Y., Cui S., Zheng Q., Lin Y., Cui Q., Xie Y., Zeng Y., Zhang C., Li Y. (2025). Methylcobalamin protects against liver failure via engaging gasdermin E. Nat. Commun..

[45] Voronina M.V., Frolova A.S., Kolesova E.P., Kuldyushev N.A., Parodi A., Zamyatnin Jr A.A. (2024). The Intricate Balance between Life and Death: ROS, cathepsins, and their interplay in cell death and autophagy. Int. J. Mol. Sci..

[46] Kim D.J., Venkataraman A., Jain P.C., Wiesler E.P., DeBlasio M., Klein J., Tu S.S., Lee S., Medzhitov R., Iwasaki A. (2020). Vitamin B12 and folic acid alleviate symptoms of nutritional deficiency by antagonizing aryl hydrocarbon receptor. Proc. Natl. Acad. Sci. USA.

[47] Lauer A.A., Grimm H.S., Apel B., Golobrodska N., Kruse L., Ratanski E., Schulten N., Schwarze L., Slawik T., Sperlich S. (2022). Mechanistic link between vitamin B12 and Alzheimer’s disease. Biomolecules.

[48] Robert Harker D.M., Martinez B., Tabaac B.J. (2021). B12 deficiency and clinical presentation in the setting of nitric oxide use. Case Rep. Neurol. Med..

[49] Singh S., Chakole S., Agrawal S., Shetty N., Prasad R., Lohakare T., Wanjari M., Yelne S. (2023). A comprehensive review of upper gastrointestinal symptom management in autoimmune gastritis: Current insights and future directions. Cureus.

[50] Mouchaileh N. (2023). Vitamin B12 deficiency in older people: A practical approach to recognition and management. J. Pharm. Pract. Res..

[51] Guéant J.-L., Guéant-Rodriguez R.-M., Alpers D.H. (2022). Vitamin B12 absorption and malabsorption. Vitam. Horm..

[52] Abdelwahab O.A., Abdelaziz A., Diab S., Khazragy A., Elboraay T., Fayad T., Diab R.A., Negida A. (2024). Efficacy of different routes of vitamin B12 supplementation for the treatment of patients with vitamin B12 deficiency: A systematic review and network meta-analysis. Ir. J. Med. Sci..

[53] Froese D.S., Gravel R.A. (2010). Genetic disorders of vitamin B12 metabolism: Eight complementation groups–eight genes. Expert Rev. Mol. Med..

[54] Watkins D., Rosenblatt D.S. (2016). Lessons in biology from patients with inherited disorders of vitamin B12 and folate metabolism. Biochimie.

[55] Forny P., Hörster F., Ballhausen D., Chakrapani A., Chapman K.A., Dionisi-Vici C., Dixon M., Grünert S.C., Grunewald S., Haliloglu G. (2021). Guidelines for the diagnosis and management of methylmalonic acidaemia and propionic acidaemia: First revision. J. Inherit. Metab. Dis..

[56] Rutsch F., Gailus S., Miousse I.R., Suormala T., Sagné C., Toliat M.R., Nürnberg G., Wittkampf T., Buers I., Sharifi A. (2009). Identification of a putative lysosomal cobalamin exporter altered in the cblF defect of vitamin B12 metabolism. Nat. Genet..

[57] Stabler S.P. (2013). Vitamin B12 deficiency. N. Engl. J. Med..

[58] Green R. (2017). Vitamin B12 deficiency from the perspective of a practicing hematologist. Blood J. Am. Soc. Hematol..

[59] Green R., Allen L.H., Bjørke-Monsen A.-L., Brito A., Guéant J.-L., Miller J.W., Molloy A.M., Nexo E., Stabler S., Toh B.-H. (2017). Vitamin B12 deficiency. Nat. Rev. Dis. Primers.

[60] Steinberg S.E., Fonda S., Campbell C.L., Hillman R.S. (1983). Cellular abnormalities of folate deficiency. Br. J. Haematol..

[61] Didangelos T., Karlafti E., Kotzakioulafi E., Kontoninas Z., Margaritidis C., Giannoulaki P., Kantartzis K. (2020). Efficacy and safety of the combination of superoxide dismutase, alpha lipoic acid, vitamin B12, and carnitine for 12 months in patients with diabetic neuropathy. Nutrients.

[62] Jin H.Y., Lee K.A., Kim Y.J., Gwak I.S., Park T.S., Yeom S.W., Kim J.S. (2023). Bidirectional association between diabetic peripheral neuropathy and vitamin B12 deficiency: Two longitudinal 9-year follow-up studies using a national sample cohort. Prim. Care Diabetes.

[63] Wang J., Wu Y., Liu S., Lin Y., Lu P. (2018). Vitamin B12 for herpetic neuralgia: A meta-analysis of randomised controlled trials. Complement. Ther. Med..

[64] Dhole P., Lohe V., Bhowate R., Gondivkar S.M., Kadu R., Mohod S.C., Sune R.V. (2022). Evaluation of serum vitamin B12 levels and its correlation with clinical presentation in patients with trigeminal neuralgia. J. Oral Biol. Craniofacial Res..

[65] Scalabrino G. (2009). The multi-faceted basis of vitamin B12 (cobalamin) neurotrophism in adult central nervous system: Lessons learned from its deficiency. Prog. Neurobiol..

[66] Kocer B., Engur S., Ak F., Yılmaz M. (2009). Serum vitamin B12, folate, and homocysteine levels and their association with clinical and electrophysiological parameters in multiple sclerosis. J. Clin. Neurosci..

[67] Nouri A., Patel K., Montejo J., Nasser R., Gimbel D.A., Sciubba D.M., Cheng J.S. (2019). The role of vitamin B12 in the management and optimization of treatment in patients with degenerative cervical myelopathy. Glob. Spine J..

[68] Roy A., Trigun S.K. (2024). The restoration of hippocampal nerve de-myelination by methylcobalamin relates with the enzymatic regulation of homocysteine level in a rat model of moderate grade hepatic encephalopathy. J. Biochem. Mol. Toxicol..

[69] Rush E., Katre P., Yajnik C. (2014). Vitamin B12: One carbon metabolism, fetal growth and programming for chronic disease. Eur. J. Clin. Nutr..

[70] Kumari R., Agrawal A., Singh G., Dubey G. (2015). Hyperhomocysteinemia and DNA hypomethylation, reduced the monoamines synthesis in depression: A case control study. J Syst Integr Neurosci.

[71] Ziegler C., Richter J., Mahr M., Gajewska A., Schiele M.A., Gehrmann A., Schmidt B., Lesch K., Lang T., Helbig-Lang S. (2016). MAOA gene hypomethylation in panic disorder—Reversibility of an epigenetic risk pattern by psychotherapy. Transl. Psychiatry.

[72] Ziegler C., Wolf C., Schiele M.A., Feric Bojic E., Kucukalic S., Sabic Dzananovic E., Goci Uka A., Hoxha B., Haxhibeqiri V., Haxhibeqiri S. (2018). Monoamine oxidase A gene methylation and its role in posttraumatic stress disorder: First evidence from the South Eastern Europe (SEE)-PTSD study. Int. J. Neuropsychopharmacol..

[73] Zhang Y., Hodgson N.W., Trivedi M.S., Abdolmaleky H.M., Fournier M., Cuenod M., Do K.Q., Deth R.C. (2016). Decreased brain levels of vitamin B12 in aging, autism and schizophrenia. PLoS ONE.

[74] Frye R.E., Sequeira J., Quadros E., James S., Rossignol D. (2013). Cerebral folate receptor autoantibodies in autism spectrum disorder. Mol. Psychiatry.

[75] Blom J.D. (2024). Hallucinations and vitamin B12 deficiency: A systematic review. Psychopathology.

[76] Alhujaili N. (2023). Catatonia and vitamin B12 deficiency-A hidden cause? A review article. Eur. Rev. Med. Pharmacol. Sci..

[77] Temova Rakuša Ž., Roškar R., Hickey N., Geremia S. (2022). Vitamin B12 in foods, food supplements, and medicines—A review of its role and properties with a focus on its stability. Molecules.

[78] Castelli M.C., Friedman K., Sherry J., Brazzillo K., Genoble L., Bhargava P., Riley M.G.I. (2011). Comparing the efficacy and tolerability of a new daily oral vitamin B12 formulation and intermittent intramuscular vitamin B12 in normalizing low cobalamin levels: A randomized, open-label, parallel-group study. Clin. Ther..

[79] Rozgony N.R., Fang C., Kuczmarski M.F., Bob H. (2010). Vitamin B12 deficiency is linked with long-term use of proton pump inhibitors in institutionalized older adults: Could a cyanocobalamin nasal spray be beneficial?. J. Nutr. Elder..

[80] Gaby S.K. (1990). Vitamin Intake and Health: A Scientific Review.

[81] Han S.J., Lee H.T. (2019). Mechanisms and therapeutic targets of ischemic acute kidney injury. Kidney Res. Clin. Pract..

[82] Karbalaei-Saleh S., Yousefi S., Honarvar M. (2024). Optimization of vitamin B12 nano-emulsification and encapsulation using spontaneous emulsification. Food Sci. Biotechnol..

[83] Mohamad S.A., Abdelkader H., Elrehany M., Mansour H.F. (2019). Vitamin B12 buccoadhesive tablets: Auspicious non-invasive substitute for intra muscular injection: Formulation, in vitro and in vivo appraisal. Drug Dev. Ind. Pharm..

[84] Sawant R.B., Nikam S.P., Roy A., Kumar A., Mohammed O.A., Sharma K., Rai A.K., Roy A., Gaur A., Verma R. (2024). Nanocarriers for nutraceutical delivery: A miniaturized revolution in health. Nano-Struct. Nano-Objects.

[85] Kuznetsova E.V., Vantsyan M.A., Kalinin K.T., Konshina E.A., Sedush N.G., Bakirov A.V., Streltsov D.R., Bukreeva T.V., Chvalun S.N. (2025). Poly (D, L-lactide-co-glycolide) Nanoparticles Modified by Layer-by-Layer Adsorption of Polyethyleneimine and Dextran Sulfate for Cyanocobalamin Embedding. BioNanoScience.

[86] Karoshi V.R., Nallamuthu I., Anand T. (2024). Co-encapsulation of vitamins B6 and B12 using zein/gum arabic nanocarriers for enhanced stability, bioaccessibility, and oral bioavailability. J. Food Sci..

[87] Bajaj S.R., Marathe S.J., Singhal R.S. (2021). Co-encapsulation of vitamins B12 and D3 using spray drying: Wall material optimization, product characterization, and release kinetics. Food Chem..

[88] Coelho S.C., Laget S., Benaut P., Rocha F., Estevinho B.N. (2021). A new approach to the production of zein microstructures with vitamin B12, by electrospinning and spray drying techniques. Powder Technol..

[89] Marchianò V., Matos M., Serrano E., Álvarez J.R., Marcet I., Blanco-López M.C., Gutiérrez G. (2022). Lyophilised nanovesicles loaded with vitamin B12. J. Mol. Liq..

[90] Salsabila D.M. (2020). Defisiensi vitamin B12 dan gangguan neurologis. J. Med. Hutama.

[91] Ljungblad U.W., Astrup H., Mørkrid L., Hager H.B., Lindberg M., Eklund E.A., Bjørke-Monsen A.-L., Rootwelt T., Tangeraas T. (2022). Breastfed infants with spells, tremor, or irritability: Rule out vitamin B12 deficiency. Pediatr. Neurol..

[92] Sahu P., Thippeswamy H., Chaturvedi S.K. (2022). Neuropsychiatric manifestations in vitamin B12 deficiency. Vitamins and Hormones.

[93] Demir N., Koc A., Üstyol L., Peker E., Abuhandan M. (2013). Clinical and neurological findings of severe vitamin B12 deficiency in infancy and importance of early diagnosis and treatment. J. Paediatr. Child Health.

[94] Pavlov C.S., Damulin I.V., Shulpekova Y.O., Andreev E.A. (2019). Neurological disorders in vitamin B12 deficiency. Ther. Arch..

[95] Kripps K.A., Sremba L., Larson A.A., Van Hove J.L., Nguyen H., Wright E.L., Mirsky D.M., Watkins D., Rosenblatt D.S., Ketteridge D. (2022). Methionine synthase deficiency: Variable clinical presentation and benefit of early diagnosis and treatment. J. Inherit. Metab. Dis..

[96] Kruman I.I., Culmsee C., Chan S.L., Kruman Y., Guo Z., Penix L., Mattson M.P. (2000). Homocysteine elicits a DNA damage response in neurons that promotes apoptosis and hypersensitivity to excitotoxicity. J. Neurosci..

[97] Suryavanshi U., Angadi K.K., Reddy V.S., Reddy G.B. (2024). Neuroprotective role of vitamin B12 in streptozotocin-induced type 1 diabetic rats. Chem. Biol. Interact..

[98] Mehrdad J., Leila E., Emsehgol N. (2023). The effect of vitamin B12 on synaptic plasticity of hippocampus in Alzheimer’s disease model rats. Int. J. Neurosci..

[99] Blaise S.A., Nédélec E., Schroeder H., Alberto J.-M., Bossenmeyer-Pourié C., Guéant J.-L., Daval J.-L. (2007). Gestational vitamin B deficiency leads to homocysteine-associated brain apoptosis and alters neurobehavioral development in rats. Am. J. Pathol..

[100] Andrade S., Ramalho M.J., Loureiro J.A., Pereira M.C. (2022). Transferrin-functionalized liposomes loaded with vitamin VB12 for Alzheimer’s disease therapy. Int. J. Pharm..

[101] Guler E., Yekeler H.B., Parviz G., Aydin S., Asghar A., Dogan M., Ikram F., Kalaskar D.M., Cam M.E. (2024). Vitamin B12-loaded chitosan-based nanoparticle-embedded polymeric nanofibers for sublingual and transdermal applications: Two alternative application routes for vitamin B12. Int. J. Biol. Macromol..

[102] Yang C.-W., Hsu H.-Y., Lee Y.-Z., Lee S.-J. (2024). Vitamin B12 inhibits peptidylarginine deiminases and ameliorates rheumatoid arthritis in CAIA mice. Biochem. Biophys. Res. Commun..

[103] Belal A., Mahmoud R., Taha M., Halfaya F.M., Hassaballa A., Elbanna E.S., Khaled E., Farghali A., Abo El-Ela F.I., Mahgoub S.M. (2023). Therapeutic potential of zeolites/vitamin B12 nanocomposite on complete Freund’s adjuvant-induced arthritis as a bone disorder: In vivo study and bio-molecular investigations. Pharmaceuticals.

[104] Belal A., Mahmoud R., Mohamed E.E., Farghali A., Abo El-Ela F.I., Gamal A., Halfaya F.M., Khaled E., Farahat A.A., Hassan A.H. (2023). A novel hydroxyapatite/vitamin B12 nanoformula for treatment of bone damage: Preparation, characterization, and anti-arthritic, anti-inflammatory, and antioxidant activities in chemically induced arthritic rats. Pharmaceuticals.

[105] Brescoll J., Daveluy S. (2015). A review of vitamin B12 in dermatology. Am. J. Clin. Dermatol..

[106] Elgharably N., Al Abadie M., Al Abadie M., Ball P.A., Morrissey H. (2022). Vitamin B group levels and supplementations in dermatology. Dermatol. Rep..

[107] Iapichino M., Maibach H., Stoeber B. (2023). Quantification methods comparing in vitro and in vivo percutaneous permeation by microneedles and passive diffusion. Int. J. Pharm..

[108] Guillot A.J., Jornet-Mollá E., Landsberg N., Milián-Guimerá C., Montesinos M.C., Garrigues T.M., Melero A. (2021). Cyanocobalamin ultraflexible lipid vesicles: Characterization and in vitro evaluation of drug-skin depth profiles. Pharmaceutics.

[109] Guillot A.J., Merino-Gutierrez P., Bocchino A., O’Mahony C., Giner R.M., Recio M.C., Garrigues T.M., Melero A. (2022). Exploration of Microneedle-assisted skin delivery of cyanocobalamin formulated in ultraflexible lipid vesicles. Eur. J. Pharm. Biopharm..

[110] Guillot A.J., Martínez-Navarrete M., Giner R.M., Recio M.C., Santos H.A., Cordeiro A.S., Melero A. (2024). Cyanocobalamin-loaded dissolving microneedles diminish skin inflammation in vivo. J. Control. Release.

[111] Farzanfar S., Kouzekonan G.S., Mirjani R., Shekarchi B. (2020). Vitamin B12-loaded polycaprolacton/gelatin nanofibrous scaffold as potential wound care material. Biomed. Eng. Lett..

[112] Bhattacharyya S.K., Nandi S., Dey T., Ray S.K., Mandal M., Das N.C., Banerjee S. (2022). Fabrication of a vitamin B12-loaded carbon dot/mixed-ligand metal organic framework encapsulated within the gelatin microsphere for pH sensing and in vitro wound healing assessment. ACS Appl. Bio Mater..

[113] Macri A., Scanarotti C., Bassi A.M., Giuffrida S., Sangalli G., Traverso C.E., Iester M. (2015). Evaluation of oxidative stress levels in the conjunctival epithelium of patients with or without dry eye, and dry eye patients treated with preservative-free hyaluronic acid 0.15% and vitamin B12 eye drops. Graefe’s Arch. Clin. Exp. Ophthalmol..

[114] Romano M.R., Biagioni F., Carrizzo A., Lorusso M., Spadaro A., Ferrari T.M., Vecchione C., Zurria M., Marrazzo G., Mascio G. (2014). Effects of vitamin B12 on the corneal nerve regeneration in rats. Exp. Eye Res..

[115] Yang J., Liu Y., Xu Y., Li X., Fu J., Jiang X., Chou Y., Ma J., Hao R., Zhang R. (2019). A new approach of ocular nebulization with vitamin B12 versus oxytocin for the treatment of dry eye disease: An in vivo confocal microscopy study. Drug Des. Dev. Ther..

[116] Meng T., Kulkarni V., Simmers R., Brar V., Xu Q. (2019). Therapeutic implications of nanomedicine for ocular drug delivery. Drug Discov. Today.

[117] Mohamad S.A., Alaaeldin E., Abdallah R.M., Mansour H.F. (2021). A New Approach for Dry Eye Management By Mucoadhesive In situ Gel of Vitamin B12: Formulation, In vitro and In vivo Assessment. AAPS PharmSciTech.

[118] Morales-Gutierrez J., Díaz-Cortés S., Montoya-Giraldo M.A., Zuluaga A.F. (2020). Toxicity induced by multiple high doses of vitamin B12 during pernicious anemia treatment: A case report. Clin. Toxicol..

[119] Stachura A., Banaszek Ł., Jurkin K., Święcicki Ł. (2024). Vitamin B12 overdose may trigger the onset of mixed-state bipolar disorder: A case report. Bipolar Disord..

[120] Koprivica M., Bjelanovic J. (2021). HYPERVITAMINOSIS B12. Med. J./Med. Časopis.

[121] Forgie A.J., Pepin D.M., Ju T., Tollenaar S., Sergi C.M., Gruenheid S., Willing B.P. (2023). Over supplementation with vitamin B12 alters microbe-host interactions in the gut leading to accelerated Citrobacter rodentium colonization and pathogenesis in mice. Microbiome.

[122] Takahata Y., Nishizawa A., Kojima I., Yamanishi M., Toraya T. (1995). Synthesis, properties and microbiological activity of hydrophobic derivatives of vitamin B12. J. Nutr. Sci. Vitaminol..

[123] Garg R., Garg A. (2021). A Review on Applications of Vitamin B 12 as Therapeutic Carrier in Drug Delivery. Crit. Rev. ™ Ther. Drug Carr. Syst..

[124] Gupta Y., Ganesh N., Veer Kohli D., K Jain S. (2011). Development and characterization of doxorubicin bearing vitamin B12 coupled sterically stabilized liposomes for tumor targeting. Curr. Nanosci..

[125] Genç L., Kutlu H.M., Güney G. (2015). Vitamin B12-loaded solid lipid nanoparticles as a drug carrier in cancer therapy. Pharm. Dev. Technol..

[126] Thepphankulngarm N., Wonganan P., Sapcharoenkun C., Tuntulani T., Leeladee P. (2017). Combining vitamin B 12 and cisplatin-loaded porous silica nanoparticles via coordination: A facile approach to prepare a targeted drug delivery system. New J. Chem..

[127] Delasoie J., Rossier J., Haeni L., Rothen-Rutishauser B., Zobi F. (2018). Slow-targeted release of a ruthenium anticancer agent from vitamin B 12 functionalized marine diatom microalgae. Dalton Trans..

[128] Guo W., Deng L., Chen Z., Chen Z., Yu J., Liu H., Li T., Lin T., Chen H., Zhao M. (2019). Vitamin B12-conjugated sericin micelles for targeting CD320-overexpressed gastric cancer and reversing drug resistance. Nanomedicine.

[129] Othayoth R., Khatri K., Gadicherla R., Kodandapani S., Botlagunta M. (2023). Multivitamin–cisplatin encapsulated chitosan nanoparticles modulate DDX3X expression in cancer cell lines. Nano Biomed. Eng.

[130] Russell-Jones G., McTavish K., McEwan J., Thurmond B. (2012). Increasing the tumoricidal activity of daunomycin-pHPMA conjugates using vitamin B12 as a targeting agent. J. Cancer Res. Updates.

[131] Ruiz-Sánchez P., König C., Ferrari S., Alberto R. (2011). Vitamin B 12 as a carrier for targeted platinum delivery: In vitro cytotoxicity and mechanistic studies. JBIC J. Biol. Inorg. Chem..

[132] Mehder R., de la Torre-Rubio E., de la Cueva-Alique I., O’Malley C., Pérez-Redondo A., Gude L., Royo E., Ronconi L. (2024). Fluorescent Vitamin B12–Platinum (II) Derivatives as Potential Metallotheranostic Agents for the Treatment and Imaging of Tumors. Inorganics.

[133] Collins D.A. (2019). Imaging cobalamin uptake within malignant breast tumors in vivo. Mol. Imaging Biol..

[134] Boss S.D., Ametamey S.M. (2020). Development of folate receptor− targeted PET radiopharmaceuticals for tumor imaging—A bench-to-bedside journey. Cancers.

[135] Sahin M.C., Sanli S. (2023). Vitamin-based radiopharmaceuticals for tumor imaging. Med. Oncol..

[136] Shahrokhi P., Farahani A.M., Tamaddondar M. (2022). Radiolabeled vitamins as the potential diagnostic probes for targeted tumor imaging. Bioorganic Chem..

[137] Leischner C., Marongiu L., Piotrowsky A., Niessner H., Venturelli S., Burkard M., Renner O. (2023). Relevant membrane transport proteins as possible gatekeepers for effective pharmacological ascorbate treatment in cancer. Antioxidants.

[138] Aggeletopoulou I., Kalafateli M., Geramoutsos G., Triantos C. (2024). Recent Advances in the Use of Vitamin D Organic Nanocarriers for Drug Delivery. Biomolecules.

[139] Atoum M.F., Alzoughool F.E., Al-Mazaydeh Z.A., Rammaha M.S., Tahtamouni L.H. (2022). Vitamin B12 enhances the antitumor activity of 1, 25-dihydroxyvitamin D3 via activation of caspases and targeting actin cytoskeleton. Tumor Biol..

[140] Solovieva M., Shatalin Y., Fadeev R., Krestinina O., Baburina Y., Kruglov A., Kharechkina E., Kobyakova M., Rogachevsky V., Shishkova E. (2020). Vitamin B12b enhances the cytotoxicity of diethyldithiocarbamate in a synergistic manner, inducing the paraptosis-like death of human larynx carcinoma cells. Biomolecules.

[141] Obeid R. (2022). High plasma vitamin B12 and cancer in human studies: A scoping review to judge causality and alternative explanations. Nutrients.

[142] Urbanski G., Hamel J.-F., Prouveur B., Annweiler C., Ghali A., Cassereau J., Lozac’h P., Lavigne C., Lacombe V. (2020). Strength of the association of elevated vitamin B12 and solid cancers: An adjusted case-control study. J. Clin. Med..

[143] Luu H.N., Wang R., Jin A., Koh W.-P., Yuan J.-M. (2021). The association between dietary vitamin B12 and lung cancer risk: Findings from a prospective cohort study. Eur. J. Cancer Prev..

[144] Stepanova M., Levit M., Egorova T., Nashchekina Y., Sall T., Demyanova E., Guryanov I., Korzhikova-Vlakh E. (2024). Poly (2-Deoxy-2-Methacrylamido-D-Glucose)-Based complex conjugates of colistin, deferoxamine and vitamin B12: Synthesis and biological evaluation. Pharmaceutics.

[145] Wald E.L., Badke C.M., Hintz L.K., Spewak M., Sanchez-Pinto L.N. (2022). Vitamin therapy in sepsis. Pediatr. Res..

[146] Dubashynskaya N.V., Bokatyi A.N., Gasilova E.R., Dobrodumov A.V., Dubrovskii Y.A., Knyazeva E.S., Nashchekina Y.A., Demyanova E.V., Skorik Y.A. (2022). Hyaluronan-colistin conjugates: Synthesis, characterization, and prospects for medical applications. Int. J. Biol. Macromol..

[147] Singh A., Yadagiri G., Parvez S., Singh O.P., Verma A., Sundar S., Mudavath S.L. (2020). Formulation, characterization and in vitro anti-leishmanial evaluation of amphotericin B loaded solid lipid nanoparticles coated with vitamin B12-stearic acid conjugate. Mater. Sci. Eng. C.

[148] Sarhadi S., Moosavian S.A., Mashreghi M., Rahiman N., Golmohamadzadeh S., Tafaghodi M., Sadri K., Chamani J., Jaafari M.R. (2022). B12-functionalized PEGylated liposomes for the oral delivery of insulin: In vitro and in vivo studies. J. Drug Deliv. Sci. Technol..

[149] Maiorova L.A., Gromova O.A., Torshin I.Y., Bukreeva T.V., Pallaeva T.N., Nabatov B.V., Dereven’kov I.A., Bobrov Y.A., Bykov A.A., Demidov V.I. (2024). Nanoparticles of nucleotide-free analogue of vitamin B12 formed in protein nanocarriers and their neuroprotective activity in vivo. Colloids Surf. B: Biointerfaces.

[150] Kolesova E.P., Egorova V.S., Syrocheva A.O., Frolova A.S., Kostyushev D., Kostyusheva A., Brezgin S., Trushina D.B., Fatkhutdinova L., Zyuzin M. (2023). Proteolytic resistance determines albumin nanoparticle drug delivery properties and increases cathepsin B, D, and G expression. Int. J. Mol. Sci..

